# An Endogenous Foamy-like Viral Element in the Coelacanth Genome

**DOI:** 10.1371/journal.ppat.1002790

**Published:** 2012-06-28

**Authors:** Guan-Zhu Han, Michael Worobey

**Affiliations:** Department of Ecology and Evolutionary Biology, University of Arizona, Tucson, Arizona, United States of America; Fred Hutchinson Cancer Research Center, United States of America

## Abstract

Little is known about the origin and long-term evolutionary mode of retroviruses. Retroviruses can integrate into their hosts' genomes, providing a molecular fossil record for studying their deep history. Here we report the discovery of an endogenous foamy virus-like element, which we designate ‘coelacanth endogenous foamy-like virus’ (CoeEFV), within the genome of the coelacanth (*Latimeria chalumnae*). Phylogenetic analyses place CoeEFV basal to all known foamy viruses, strongly suggesting an ancient ocean origin of this major retroviral lineage, which had previously been known to infect only land mammals. The discovery of CoeEFV reveals the presence of foamy-like viruses in species outside the Mammalia. We show that foamy-like viruses have likely codiverged with their vertebrate hosts for more than 407 million years and underwent an evolutionary transition from water to land with their vertebrate hosts. These findings suggest an ancient marine origin of retroviruses and have important implications in understanding foamy virus biology.

## Author Summary

The deep history of retroviruses is still obscure. Retroviruses can leave integrated copies within their hosts' genomes, providing a fossil record for studying their long-term evolution. Endogenous forms of foamy viruses, complex retroviruses known to infect only mammalian species, appear to be extremely rare, so far found only in sloths and the aye-aye. Here, we report the discovery of endogenous foamy virus-like insertions within the genome of a so-called ‘living fossil’, the coelacanth (*Latimeria chalumnae*). We provide evidence suggesting that foamy viruses and their hosts share a coevolutionary history of more than 407 million years, and that foamy viruses accompanied their vertebrate hosts on the evolutionary transition from water to land. These findings indicate that the retroviruses originated in the primeval ocean millions of years ago.

## Introduction

Foamy viruses are complex retroviruses thought exclusively to infect mammalian species, including cats, cows, horses, and non-human primates [1]. Although human-specific foamy viruses have not been found, humans can be naturally infected by foamy viruses of non-human primate origin [2]–[4]. Comparing the phylogenies of simian foamy viruses (SFVs) and Old World primates suggests they co-speciated with each other for more than 30 million years [5]. Retroviruses can invade their hosts' genomes in the form of endogenous retroviral elements (ERVs), providing ‘molecular fossils’ for studying the deep history of retroviruses and the long-term arms races between retroviruses and their hosts [6], [7]. Although ERVs are common components of vertebrate genomes (for example, ERVs constitute around 8% of the human genome) [8], germline invasion by foamy virus seems to be very rare [9], [10]. To date, endogenous foamy virus-like elements have been discovered only within the genomes of sloths (SloEFV) [9] and the aye-aye (PSFVaye) [10]. The discovery of SloEFV extended the co-evolutionary history between foamy viruses and their mammal hosts at least to the origin of placental mammals [9]. However, the ultimate origin of foamy virus and other retroviruses remains elusive.

The continual increase in eukaryotic genome-scale sequence data is facilitating the discovery of additional ERVs, providing important insights into the origin and long-term evolution of this important lineage of viruses. In this study, we report the discovery and analysis of an endogenous foamy virus-like element in the genome of the coelacanth (*Latimeria chalumnae*), which we designate ‘coelacanth endogenous foamy-like virus’ (CoeEFV). The discovery CoeEFV offers unique insights into the origin and evolution of foamy viruses and the retroviruses as a whole.

## Results/Discussion

### Discovery of foamy virus-like elements within the genome of coelacanth

**Figure 1:**
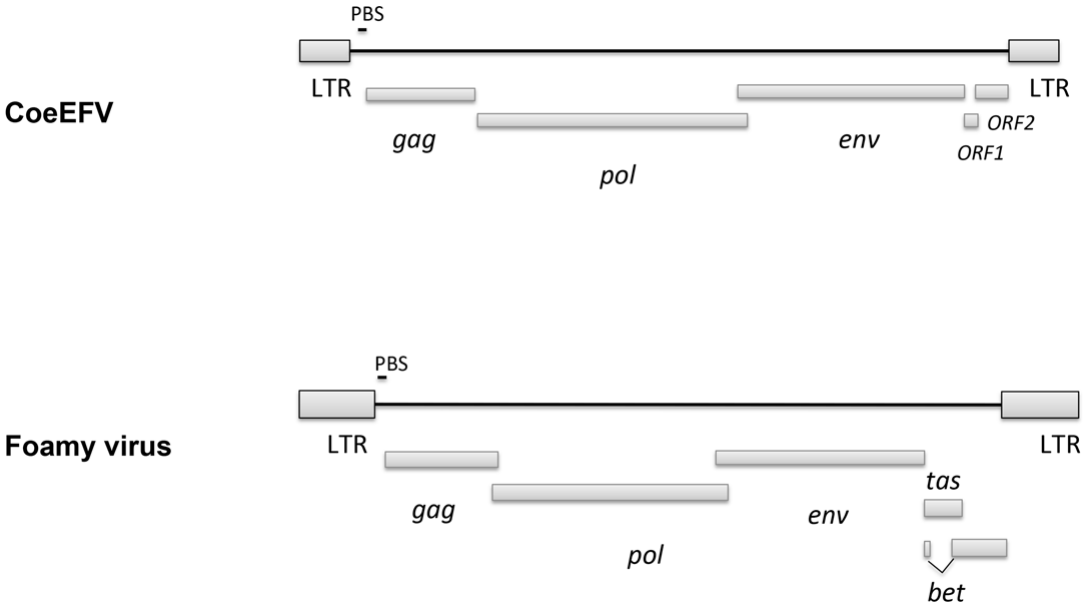
Comparison of the genome structures between CoeEFV and typical exogenous foamy virus. LTR, long-terminal repeat; PBS, primer-binding site.

**Figure 2:**
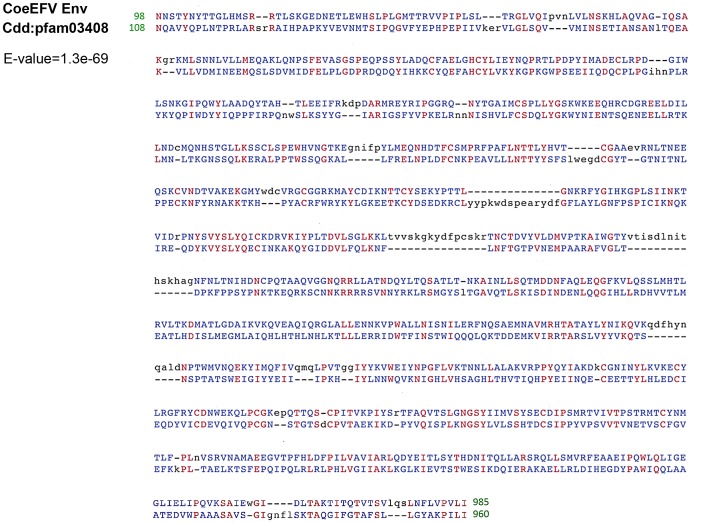
Conserved domain alignment of the CoeEFV Env protein and foamy virus envelope protein domain (pfam03408). Numbers refer to the position in the original CoeEFV env protein or conserved domain pfam03408. Identical amino acid residues are highlighted in red, and black and blue indicate gaps or different amino acid residues, respectively. The E-value was generated by Conserved Domain search.

We screened all available animal whole genome shotgun (WGS) sequences using the tBLASTn algorithm using the protein sequences of representative foamy viruses (Table S1) and identified several foamy virus-like insertions (Table S2 and Fig. S1) within the genome of *L. chalumnae*, one of only two surviving species of an ancient Devonian lineage of lobe-finned fishes that branched off near the root of all tetrapods [11]–[15]. There are numerous in-frame stop codons and frame-shift mutations present in these CoeEFV elements, suggesting that the CoeEFV elements might be functionally defective. Although more than 230 vertebrate genome scale sequences are currently available, endogenous foamy virus elements have been only found in the aye-aye, sloths, and coelacanth, indicating that germline invasion of foamy virus is a rare process [9], [10]. We extracted all contigs containing significant matches and reconstructed a consensus CoeEFV genomic sequence (Fig. S2). The resulting consensus genome shows recognizable and typical foamy virus characteristics (Fig. 1). Its genome has long terminal repeat (LTR) sequences at both 5′ and 3′ ends and encodes the three main open reading frames (ORFs), *gag*, *pol*, and *env*, in positions similar to those of exogenous foamy viruses (Fig. 1). Two additional putative ORFs were found at positions similar to known foamy virus accessory genes but exhibit no significant similarity (Fig. 1). Notably, we found that the Env protein is conserved among foamy viruses and the coelacanth virus-like element (Fig. 2). A Conserved Domain search [16] identified a conserved foamy virus envelope protein domain (pfam03408) spanning most (887 of 1016 residues) of the CoeEFV Env protein, with an E-value of 1.3×10^−69^ (Fig. 2). The CoeEFV Env protein shares no detectable similarity with other (non-foamy virus) retroviral Env proteins or with retroviral elements within available genomic sequences of other fishes, such as the zebrafish (*Danio rerio*). Hence, it provides decisive evidence that CoeEFV originated from a foamy-like virus.

To exclude the possibility that these CoeEFV elements result from laboratory contamination, we obtained a tissue sample of *L. chalumnae* and succeeded in amplifying CoeEFV insertions within the genome of *L. chalumnae* via PCR with degenerate primers designed for conserved regions of foamy virus *pol* and *env* genes.

**Figure 3:**
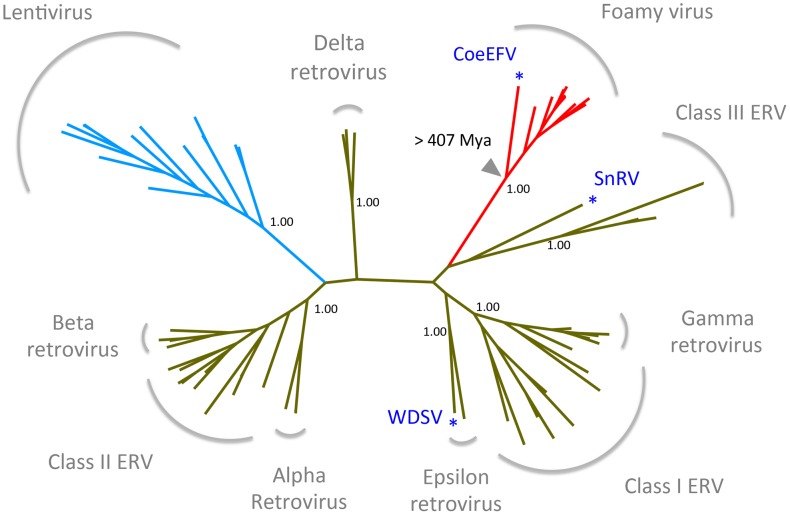
Retrovirus phylogeny. The phylogeny is the 50% majority-rule consensus tree reconstructed based on the conserved region of Pol protein of CoeEFV and various endogenous and exogenous retroviruses using MrBayes 3.1.2. Posterior probabilities are shown near the selected nodes. The foamy virus and lentivirus clades are highlighted in red and blue, respectively. The full tree and taxon labels are depicted in Fig. S2. WDSV, walleye dermal sarcoma virus; SnRV, snakehead retrovirus.

To establish the position of CoeEFV on the retrovirus phylogeny, conserved regions of the Pol protein sequences of CoeEFV and various representative endogenous and exogenous retroviruses were used to reconstruct a phylogenetic tree with a Bayesian approach. The phylogenetic tree shows that CoeEFV groups with the foamy viruses with strong support (posterior probability = 1.00; Figs. 3 and S3), confirming that CoeEFV is indeed an endogenous form of a close relative of extant foamy viruses. The discovery of CoeEFV establishes that a distinct lineage of exogenous foamy-like viruses existed (and may still exist) in species outside the Mammalia.

### CoeEFV likely invaded the coelacanth genome more than 19 million years ago

Endogenous retroviruses are likely to undergo a gradual accumulation of neutral mutations with host genome replication after endogenization [17]. To date the invasion of CoeEFV into coelacanth genome, we identified two sets of sequences, each of which arose by segmental duplication because each set of sequences shares nearly identical flanking regions (Fig. S4). The two sets contain five and two sequences, respectively. Because the divergence time of the two extant coelacanth species (*L. chalumnae* and *L. menadoensis*) is uncertain [11], it is impossible to obtain a reliable neutral evolutionary rate of coelacanth species. Nevertheless, even using the mammalian neutral evolutionary rate [18] as a proxy for the coelacanth rate, the invasion dates were conservatively estimated at 19.3 (95% highest posterior density [HPD]: 15.3–23.6) million years ago for the dataset of five sequences. For the dataset containing two sequences, the divergence between the pair is estimated to be 4.1% and the invasion time is estimated to be approximately 9.3 million years ago. Because the CoeEFV invasion almost certainly occurred earlier than the duplication events within the host genome and because the evolutionary rate of coelacanth species is thought to be lower than other vertebrate species [19], [20], the time of CoeEFV integration might much more than 19 million years. Additional phylogenetic evidence (see below) suggests that its exogenous progenitors likely infected coelacanths for hundreds of millions of years prior to the event that fossilized CoeEFV within its host's genome.

### Foamy-like viruses have likely codiverged with their vertebrate hosts for at least 407 million years

**Figure 4:**
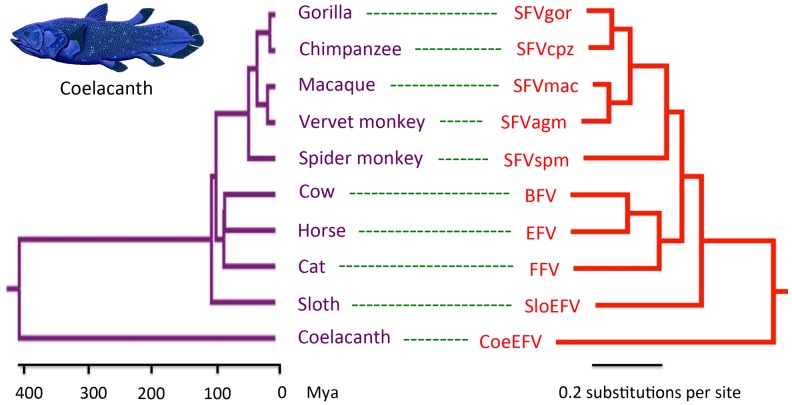
Phylogenetic congruence of foamy viruses (right) and their hosts (left). Associations between foamy viruses and their hosts are indicated by connecting lines. The scale of the host phylogeny (left) indicates millions of years. The foamy virus phylogeny (right) is the 50% majority-rule consensus tree inferred from conserved region of foamy virus and Class III retrovirus Pol protein alignment with MrBayes 3.1.2. The Bayesian phylogeny is well supported with all nodes showing posterior probability of 1.00. Branch lengths are in expected amino acid changes per site. Coelacanth image courtesy of Robbie Cada.

**Figure 5:**
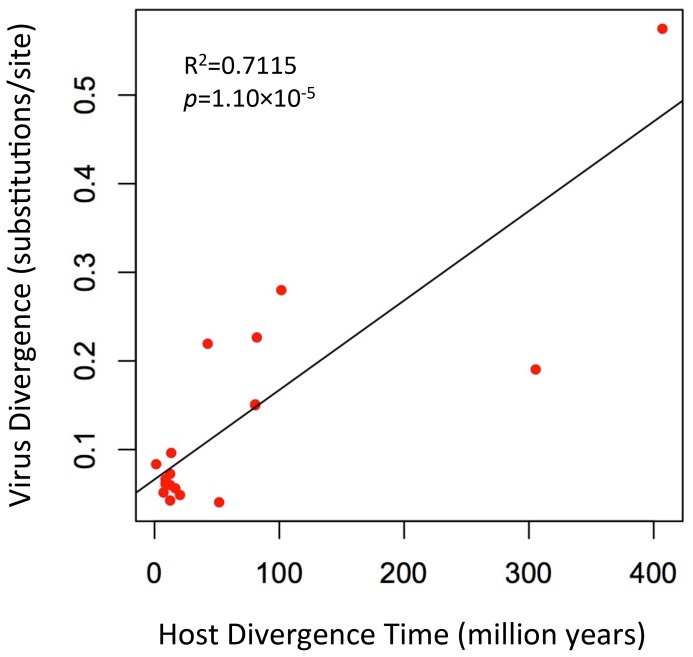
A plot of the correlation between foamy virus divergence and their vertebrate hosts' divergence times. The plot depicts host branch length (in millions of years) versus virus branch length (in expected amino acid substitutions per site) for every branch (both internal and external). The virus branch lengths are derived from the virus tree in Fig. 4.

To further evaluate the relationship of foamy viruses, we reconstructed phylogenetic trees based on the conserved region of Pol proteins of foamy viruses and Class III retroviruses, the conserved region of foamy virus Pol and Env protein concatenated alignment, and the conserved region of foamy virus Env protein alignment, respectively. The three phylogenies have the same topology in terms of foamy viruses (Figs. 4, S5, and S6). CoeEFV was positioned basal to the known foamy viruses (Fig. 4), suggesting a remarkably ancient ocean origin of foamy-like viruses: the most parsimonious explanation of this phylogenetic pattern is that foamy viruses infecting land mammals originated ultimately from a prehistoric virus circulating in lobe-finned fishes. The branching order of the three foamy virus phylogenies (Fig. 4, S5, and S6) is completely congruent with the known relationships of their hosts, and each node on the three virus trees is supported by a posterior probability of 1.0 (except the node leading to equine, bovine, and feline foamy viruses on the Env phylogeny, which is supported by a posterior probability of 0.94; Fig. S6). The common ancestor of coelacanths and tetrapods must have existed prior to the earliest known coelacanth fossil, which is 407–409 million years old [21]. The completely congruent virus topology, therefore, strongly indicates that an ancestral foamy-like virus infected this ancient animal. Crucially, the foamy viral branch lengths of the three phylogenies are highly significantly correlated with host divergence times (R^2^ = 0.7115, *p* = 1.10×10^−5^, Fig. 5; R^2^ = 0.7024, *p* = 1.41×10^−5^, Fig. S5; and R^2^ = 0.7429, *p* = 4.26×10^−6^, Fig. S6), a pattern that can reasonably be expected only if the viruses and hosts codiverged. It is worth emphasizing that we used a consensus sequence to represent CoeEFV in these analyses, so its branch length should correspond roughly to that of the exogenous virus that integrated >19 million years ago, rather than within-host mutations since that time.

There are two alternative explanations for these phylogenetic patterns. One is that the exogenous progenitor of CoeEFV is not truly the sister taxon to the mammalian foamy viruses, but a more distant relative. The robust posterior probability (1.00) placing them in the same clade and the absence of evidence for viruses or virus-like elements from other species disrupting this clade argue against this view, as does the significant similarity between the Env proteins of CoeEFV and the foamy viruses (Fig. 2). Moreover, its branch length would be difficult to explain under such a scenario. If the coelacanth foamy-like virus lineage and the mammalian foamy virus lineage did not share a most recent common ancestor in their ancestral host, why is CoeEFV neither more nor less divergent from the mammalian foamy viruses than one might expect if they did?

The other alternative to the hypothesis that these viruses have co-diverged over more than 407 million years is that they somehow moved, in more recent times, from terrestrial hosts to sarcopterygian hosts that inhabited the deep sea, and that the similarity of the coelacanth virus to the mammalian viruses is due to cross-species (in fact cross-class) transmission, rather than shared history. However, as illustrated by the significant correlation between host divergence times and viral distances (Figs. 5, S5, and S6), the long branches leading to CoeEFV and the clade of mammal foamy viruses suggest the virus had already circulated in vertebrates for an extremely long time before the origin of mammal foamy virus. Given that there is strong evidence that placental mammals were already being infected with foamy viruses by about 100 million years ago [9], the distinctness of the coelacanth virus suggests that it would have to have crossed from some other unidentified host, one whose foamy-like virus was already hundreds of millions of years divergent from the mammalian viruses. This seems highly unlikely. Although cross-species transmission of SFVs has been observed [2]–[5], [22], foamy viruses seem to mainly follow a pattern of co-diversification with their hosts [5], [9]. If one accepts that the endogenous foamy viruses within the genomes sloths indicate more than 100 million years of host-virus co-divergence, it seems plausible that CoeEFV extends that timeline by an additional 300 million years.

Moreover, the habitat isolation of the coelacanth and terrestrial vertebrates would have provided limited opportunities for direct transfer of foamy viruses to coelacanths. Taken together, these lines of evidence strongly suggest that foamy viruses and their vertebrate hosts have codiverged for more than 407 million years, and that foamy viruses underwent a remarkable evolutionary transition from water to land simultaneously with the conquest of land by their vertebrate hosts.

Our analyses provide compelling evidence for the existence of retroviruses going back at least to the Early Devonian. This is the oldest estimate, to our knowledge, for any group of viruses, significantly older than the previous estimates for hepadnaviruses (19 million years) [23] and large dsDNA viruses of insects (310 million years) [24]. Although highly cytopathic in tissue culture, foamy viruses do not seem to cause any recognizable disease in their natural hosts [1], [25], [26]. Such long-term virus-host coevolution may help explain the low pathogenicity of foamy viruses. The fact that the Env is well conserved between CoeEFV and foamy viruses is consistent with the fact that these viruses are asymptomatic and mainly co-evolve with their hosts in a relatively conflict-free relationship. It is easy to imagine that previously overlooked examples of such a non-pathogenic virus may yet be found in hosts that fill in some of the gaps in the phylogeny, namely amphibians, reptiles, and birds. It will be of interest to screen these hosts, but also various fish species, for evidence of exogenous and/or endogenous foamy-like viruses.

### An ancient marine origin of retroviruses

Dating analyses provide the clearest evidence for when and where retroviruses originated. There is strong evidence that foamy viruses shared a common, exogenous retroviral ancestor more than 400 million years ago (since Env was present in both terrestrial and marine lineages). The discovery of endogenous lentiviruses demonstrates that lentiviruses, a distinct retroviral lineage that includes HIV, are also millions of years old [27]–[30]. Foamy viruses and lentiviruses share a distantly related ancestor (Figs. 3, S3) and the foamy virus clade alone almost certainly accounts for more than 407 million years of retroviral evolution. It follows that the origin of at least some retroviruses is older than 407 million years ago. As with the coelacanth lineage in the foamy virus clade, we found that retroviruses of fishes occupy the most basal positions within both the Class I and Class III retroviral clades (walleye dermal sarcoma virus (WSDV) and snakehead retrovirus (SnRV), respectively, blue asterisks), (Figs. 3, S3). This pattern provides additional evidence of a marine origin and long-term coevolution of these major retroviral lineages. However, to be specific, the phylogenetic reconstruction in Fig. 3 reflects the history of only of the Pol protein, not a comprehensive history of retroviral genomic evolution. Nevertheless, our analyses support a very ancient marine origin of retroviruses.

## Materials and Methods

### Screening and consensus genome construction

All available animal whole genome shotgun (WGS) sequences from GenBank were screened for endogenous foamy viruses using the tBLASTn algorithm and the protein sequences of representative exogenous and endogenous foamy viruses (Table S1). Sequences highly similar to foamy virus proteins discovered within the coelacanth WGS were aligned to generate a CoeEFV consensus genome. Conserved domains were identified using CD-Search service [16].

### PCR amplification and cloning of CoeEFV

Ethanol preserved *Latimeria chalumnae* tissue sample was obtained from Ambrose Monell Cryo Collection (AMCC) at the American Museum of Natural History, New York. Genomic DNA was extracted using the DNeasy tissue kit (QIAGEN, MD) following the manufacturer's instructions. Amplification of ∼680 bp *gag* gene and ∼650 bp *env* gene fragments was performed with the degenerate primer pairs, FVpol-F (5′-AACAGTGYCTYGACCMAACC-3′) and FVpol-R (5′-TAGTGAGCGCTGCTTTGAGA-3′), FVenv-F (5′-CTGGGGATGACAAYCAGAGT-3′) and FVenv-R (5′-CCACTCRGGAGAGAGGCAAC-3′). PCR was performed in 25 µl of final volume reactions with 0.1 µl Platinum Taq HiFi enzyme (Invitrogen, CA), 1 µl primer mix (10 µM each), 0.5 µl of 10 mM dNTP mixture, 1 µl of 50 mM MgSO4, 2.5 µl of 10× PCR buffer, and 1 µl of template DNA. The PCR reactions were cycled under the following conditions: initial denaturation at 94°C for 2 minutes, 45 cycles of (94°C for 15 seconds, 60°C for 60 seconds, and 72°C for 30 seconds), and final elongation at 72°C for 5 minutes. The PCR products were purified using QIAquick spin columns (QIAGEN, MD). Purified PCR products were cloned into the pGEM-T Easy vector (Promega, WI). Cloned products were sequenced by the University of Arizona Genetics Core with an Applied Biosystems 3730XL DNA Analyzer. The sequences have been deposited in GenBank (Accession Nos. JX006240-JX006251).

### Phylogenetic analysis

All protein sequences were aligned using Clustal Omega [31]. Gblocks 0.91b was used to eliminate ambiguous regions and extract conserved regions from the alignments [32]. To determine the phylogenetic relationship between CoeEFV and other retrovirus, we reconstructed a phylogeny based on the conserved region of Pol proteins of CoeEFV and various representative exogenous and endogenous retroviruses (Table S1; Dataset S1). To further evaluate the relationship and divergence of foamy viruses, the conserved region of the foamy viruses and Class III endogenous retroviruses Pol protein (Dataset S2), the conserved region of foamy virus Pol and Env protein concatenated alignment (Dataset S3), and the conserved region of foamy virus Env protein alignment (Dataset S4) were used to infer phylogenetic trees. We were unable to discern positional homology for the first 143 residues of the Pol protein with reasonable certainty. These regions were excluded from all subsequent analyses. All the phylogenetic analyses were performed with MrBayes 3.1.2 [33] using 1,000,000 generations in four chains, sampling posterior trees every 100 generations. The rtREV amino acid substitution model [34] was used. The first 25% of the posterior trees were discarded. MCMC convergence was indicated by an effective sample size >300 as calculated in the program Tracer v1.5.

### Host-virus branch length analysis

For the phylogenetic tree based on the foamy viruses and Class III endogenous retroviruses Pol protein, Class III endogenous retroviruses were used to root the foamy viral phylogeny (Fig. 4). Because there is no obvious outgroup for foamy virus Env protein, we rooted the phylogenetic trees inferred from foamy virus Pol and Env concatenated alignment and Env alignment using midpoint method (Figs. S5 and S6). Because the topologies of the host and virus trees were identical for the foamy viruses (Figs. 4, S5, and S6), we were able to plot host branch length (in millions of years) versus virus branch length (in expected amino acid substitutions per site) for every branch (both internal and external). The vertebrate host divergence times are based on references [21], [35], and [36].

### Dating analysis

The nucleotide sequences were aligned using MUSCLE [37]. To estimate the age of the CoeEFV invasion, we identified two sets of sequences, which contain five sequences (contig270160, contig184752, contig185880, contig245863, and contig236769) (Dataset S5) and two sequences (contig243355 and contig219087) (Dataset S6). Sharing the same flanking region, each set of sequences arose from segmental duplication. I) For the dataset of five sequences: the best-fitting model of nucleotide substitution was determined using jModelTest [38]. The typical mammal neutral evolutionary rate (2.2×10^−9^ substitutions per site per year, standard deviation = 0.1×10^−9^) was used as the rate prior [18]. The HKY substitution model was used. BEAST v1.6.1 (http://beast.bio.ed.ac.uk) was employed for Bayesian MCMC analysis with a strict clock model [39] and Yule model of speciation. MCMC chains were run for 100 million steps twice to achieve adequate mixing for all parameters (effective sample size >200). Tracer v1.5 was used to summarize and analyze the resulting posterior sample. II) For the dataset of two sequences: we calibrated the genetic distance between the pair based on the Kimura two-parameter model, in which transitions and transversions are treated separately.

## Supporting Information

Dataset S1 The conserved region of Pol proteins of CoeEFV and various representative exogenous and endogenous retroviruses.
(TXT)

Dataset S2 The conserved region of the foamy viruses and Class III endogenous retroviruses Pol protein.
(TXT)

Dataset S3 The conserved region of foamy virus Pol and Env protein concatenated alignment.
(TXT)

Dataset S4 The conserved region of foamy virus Env protein alignment.
(TXT)

Dataset S5 The alignment of five sequences (contig270160, contig184752, contig185880, contig245863, and contig236769) used to estimate the age of the CoeEFV invasion.
(TXT)

Dataset S6 The alignment of two sequences (contig243355 and contig219087) used to estimate the age of the CoeEFV invasion.
(TXT)

Figure S1 Schematic mapping of CoEFV fragments identified in this study onto the CoeEFV consensus genome.
(PDF)

Figure S2 CoeEFV consensus genomic sequence. The ambiguous nucleotides were filled according to contig187425, contig187426, and contig178313 of coelacanth genome. The CoeEFV PBS is nearly identical to the PBS of extant foamy viruses. The CoeEFV PBS sequence is 5′-TGGCACCCAACGTGGGG-3′. The PBS sequence of human foamy virus is 5′-TGGCGCCCAACGTGGGG-3′ (Baldwin and Linial, J. Virol. 1999, 73:6387–6393). There is only one substitution.
(PDF)

Figure S3 Phylogenetic relationships among retroviruses. The phylogeny was reconstructed with the Bayesian method via MrBayes 3.1.2. The posterior probabilities are shown on the nodes. The foamy virus and lentivirus clades were highlighted in red and blue, respectively. Branch lengths are in expected amino acid changes per site.
(PDF)

Figure S4 Alignment of the two sets of sequences used for dating CoEFV invasion. Flanking sequences are shown for each sequence set with consensus genomic sequence of CoeEFV.
(PDF)

Figure S5 A) Midpoint rooted phylogenetic tree of foamy viruses. The phylogeny is the 50% majority-rule consensus tree inferred from conserved region of foamy virus Pol and Env protein concatenated alignment with MrBayes 3.1.2. Posterior probabilities are shown at the nodes. Branch lengths are in expected amino acid changes per site. B) A plot of the correlation between foamy virus divergence and their vertebrate hosts' divergence times. The virus branch lengths are derived from the virus tree in A.
(PDF)

Figure S6 A) Midpoint rooted phylogenetic tree of foamy viruses. The phylogeny is the 50% majority-rule consensus tree inferred from conserved region of foamy virus Env protein alignment with MrBayes 3.1.2. Posterior probabilities are shown at the nodes. Branch lengths are in expected amino acid changes per site. B) A plot of the correlation between foamy virus divergence and their vertebrate hosts' divergence times. The virus branch lengths are derived from the virus tree in A.
(PDF)

Table S1 The representative retrovirus sequences used for genome screening and phylogenetic reconstruction.
(PDF)

Table S2 The matching contigs identified in coelacanth genome.
(PDF)
