# Supplementary material for: An Endogenous Foamy-like Viral Element in the Coelacanth Genome
Source: PLoS Pathog. 2012 Jun 28;8(6):e1002790. doi: 10.1371/journal.ppat.1002790 (PMC3386198; doi:10.1371/journal.ppat.1002790)
Supplement: Figure S1 — Schematic mapping of CoEFV fragments identified in this study onto the CoeEFV consensus genome. (PDF) [file ppat.1002790.s007.pdf]

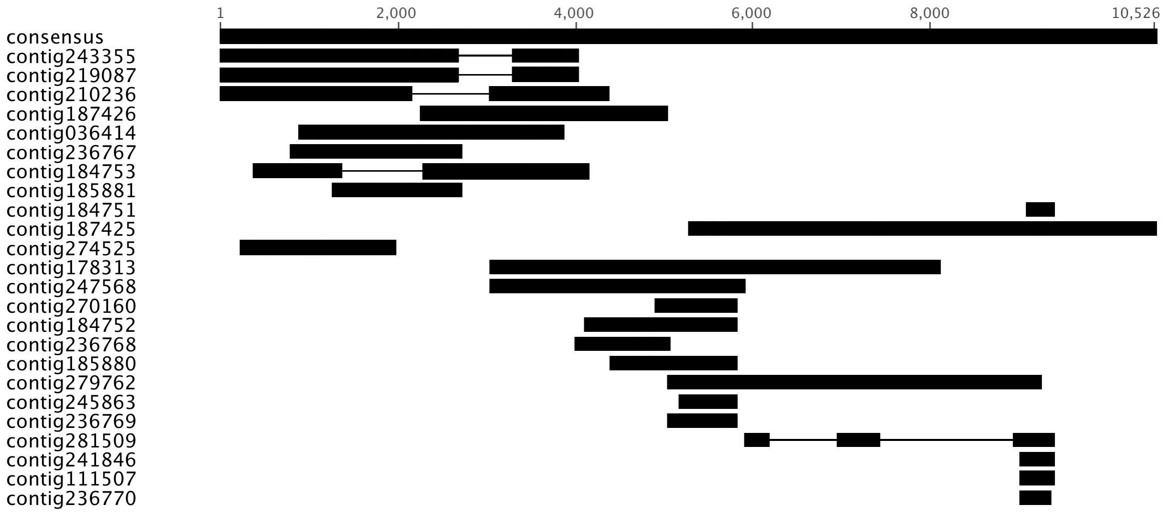

Figure S1. Schematic mapping of CoEFV fragments identified in this study onto the CoeEFV consensus genome.
