# Supplementary material for: An Endogenous Foamy-like Viral Element in the Coelacanth Genome
Source: PLoS Pathog. 2012 Jun 28;8(6):e1002790. doi: 10.1371/journal.ppat.1002790 (PMC3386198; doi:10.1371/journal.ppat.1002790)
Supplement: Figure S2 — CoeEFV consensus genomic sequence. The ambiguous nucleotides were filled according to contig187425, contig187426, and contig178313 of coelacanth genome. The CoeEFV PBS is nearly identical to the PBS of extant foamy viruses. The CoeEFV PBS sequence is 5′-TGGCACCCAACGTGGGG-3′. The PBS sequence of human foamy virus is 5′-TGGCGCCCAACGTGGGG-3′ (Baldwin and Linial, J. Virol. 1999, 73:6387–6393). There is only one substitution. (PDF) [file ppat.1002790.s008.pdf]

Figure S2. CoeEFV consensus genomic sequence. The ambiguous nucleotides were filled according to contig187425, contig187426, and contig178313 of coelacanth genome. The CoeEFV PBS is nearly identical to the PBS of extant foamy viruses. The CoeEFV PBS sequence is 5′-TGGCACCCAACGTGGGG-3′. The PBS sequence of human foamy virus is 5′-TGGCGCCCAACGTGGGG-3′ (Baldwin and Linial, J. Virol. 1999, 73:6387-6393). There is only one substitution.

```
LTR
AATGTACTAGTATAGGCTCCTTTTCCTACTGTCAGTTTCTATTGTCAGTTTAGCTTGTCATATAATTGATGTCCTAGAC
CTATTGGTATGGAAAGATCAGTAATCCTAGGTTAATTGCTTGCATGAAACTATGACGAATGTGTTACTTGCAAATAGAA
GTTCCTAGACCAGATGACCTTTTCAAAAAGGAAGTCTTGACCACAAAATGCAGTATGATTAACTACTTTGTCACAGCTG
ATCTATACAGCTGATACTGTTATGCTAATGTTTAAGGTGACAGGAAAAATGTTATTTGTTAAGTTTTGTCCTAGACTAA
AGAAATGTATAATTGTAATATCTTCCTGCATGAAGAACCTTCCTGGACATCCTGTGTAAGGACCAAGCCTAAGAGCCAG
AATATTGTATAAATACAACACCTAGGTCCATAGCATGTCAGAACGATCTTCTCTGCTGAGAGACCGAGGGTGAGAACTG
CTGATTGCAGAATCTTTGCTATTGGACTAGGTGAGCACTGTTACTATCCTTCTATATTCTTAGATTGTGAGTTCTGGCC
TGGTCCCTTTATGCTTAAGTAAGATTGATATATTGAAATTGCTAGCCTTTGTAATTTTATACTATAACCCACTCTAGAG
CTAATTGTATGCTTTATATATATCATTAATAAATATATAACATTTTAAGGACCACTCAAGCCTCTCTTTTAATTCATTA
TTACTAGAAATATATGAATCTTTATTTACTGGTGATTTAGCAATTATCAAGAGGGAGGTAAGTGGATCTACTCCTTTGA
TGTACTAAAGGATGGTTGCCTAGTACTAATTCAATGCTATCCCTATAACAGGGTAATAGTATTCAATTAGGCGGGCAAT
                  -------PBS-------
ACCACTCCACATCACAGTTGGCACCCAACGTGGGGCTTGAGTGTCCTTAATTATTTCTTACTGTTAGAATCAAAACCAT
TTCCACTATATAGTGAATGTACTGTGTATTATGGCTATTTTCGCTAACTGATCACTAGCAAATAAGAAAATAGAGTGAT
ACAATAAGTTCTCTCAGAAAGATCTGAGTTTAAGAGTTAAGCTTAACTAAATAAAAGAACAAAGCCCGAAAGTTTCCAT
 →Gag
AATGGCATTAAATATATTTAGAAGAGCATACAATGCTATAAGAAGGGGAGCTGCGGCAGCAGCCCCAAATGCAGATGAA
 M  A  L  N  I  F  R  R  A  Y  N  A  I  R  R  G  A  A  A  A  A  P  N  A  D  E
CAATTTTTGATTTGCATGATGGATTATAATGACTTTGGCATTAGAGAGTGTGTTGGTACCCTGGTATTAGATAATGCAG
Q  F  L  I  C  M  M  D  Y  N  D  F  G  I  R  E  C  V  G  T  L  V  L  D  N  A  G
GAGAGATAATTAACATCAACTGGCAGCCTAATGCTGCAGTACAAGTGGGGATGGAAATTCTTACATTTGATTATCAGGA
  E  I  I  N  I  N  W  Q  P  N  A  A  V  Q  V  G  M  E  I  L  T  F  D  Y  Q  E
ACTAGTTAATGAGGGCCTCCAGCCTCAGTTTGGGGCAGGATTAAGGAGGCATGGAAATTTAAGAGGAGTGTTATATCAC
 L  V  N  E  G  L  Q  P  Q  F  G  A  G  L  R  R  H  G  N  L  R  G  V  L  Y  H
CAAGGAGGGCCAGGATGGCAGTTTGCCCCTCTAGAACCTGCAGAATATCCCAATAATGTTCAAGACTGGCAGGTGCACT
Q  G  G  P  G  W  Q  F  A  P  L  E  P  A  E  Y  P  N  N  V  Q  D  W  Q  V  H  Y
ATGAGGCCTTAAGGAACCAATACCAGGCTCTAAGGAGAGCGGCTCCTAGGCTACAGGTCCAAGGACCAGGAGCCCCTGT
  E  A  L  R  N  Q  Y  Q  A  L  R  R  A  A  P  R  L  Q  V  Q  G  P  G  A  P  V
GCCTCAAGCACTCGAGCCCTATGGGCAATTAAATATAGGGGCTATAAAAAACATACTGGGAGATCCTCCCAATAGAGGA
 P  Q  A  L  E  P  Y  G  Q  L  N  I  G  A  I  K  N  I  L  G  D  P  P  N  R  G
GAAGATTTCCTTGATTGGTTCCAAGCCAGGCACACTCAAATAGAAACAATAACCGAGGGATACACTGCTGAACAAAGAA
E  D  F  L  D  W  F  Q  A  R  H  T  Q  I  E  T  I  T  E  G  Y  T  A  E  Q  R  R
GGCAGTTATACCAATATATCCTACCTACTACATGGGCTTCAGTTCCGATAGCTCATTGTAATAATGTACAAGCCACTTT
  Q  L  Y  Q  Y  I  L  P  T  T  W  A  S  V  P  I  A  H  C  N  N  V  Q  A  T  L
GGATCATTTGTATATCCAAATAACTGGTCAACCTCCCATTATTCGTTTAACCAAACAAATACCTCTGATAACTGCTAGC
 D  H  L  Y  I  Q  I  T  G  Q  P  P  I  I  R  L  T  K  Q  I  P  L  I  T  A  S
CAAGGAATATTGGTAGCTTATGATCATGCATACAGAGGGTTGAGAGGAGACCATGTAATGGCGTTTGAGTTAGTGCGTG
Q  G  I  L  V  A  Y  D  H  A  Y  R  G  L  R  G  D  H  V  M  A  F  E  L  V  R  E
AAGGGATTCCAATGGCAGTAAGGCTCCAAATCACTGCTGCACTGCAAGCTTTGCCAACAAATCAGGAAAGGCACGCTCA
  G  I  P  M  A  V  R  L  Q  I  T  A  A  L  Q  A  L  P  T  N  Q  E  R  H  A  Q
GTTTGCAGATATAGTAAGGCTCAATTACAATCTGGCCATGATACCATTAACTCCTACTCAGAAACAGGGATATTCCAAG
 F  A  D  I  V  R  L  N  Y  N  L  A  M  I  P  L  T  P  T  Q  K  Q  G  Y  S  K
ATACCTGGGCCCCCAAATCAAGAAGGCAATCAGAAAAAAAGGCAACAGCGCCCCCTTAAGACCCAGGGTAGAGGCCAGG
I  P  G  P  P  N  Q  E  G  N  Q  K  K  R  Q  Q  R  P  L  K  T  Q  G  R  G  Q  V
TTAAATGGACTCCTAAGCAAGAAAAAGAGGAGTCCCGGCCTCAAAGCTCCCAAGGAGCTGACAGCCGCCCAGATCCCTC
  K  W  T  P  K  Q  E  K  E  E  S  R  P  Q  S  S  Q  G  A  D  S  R  P  D  P  S
AGGAGAGATTCCTAAGAAACATAGATATGATTTGAGACCCAGGCATGATGGGCCCTGGCAGCAACCATCAGGACAAAAG
```

G E I P K K H R Y D L R P R H D G P W Q Q P S G Q K
TCTGATGGTAATCAGGGAGGACAAAAATGGTGGAAGAAAGGGCAGGACAAACAGCAACCTAAATATAAAAAAGAAGGAG
S D G N Q G G Q K W W K K G Q D K Q Q P K Y K K E G G

Gag ←

GGGATGTCAAGCATGTTAACATAAATACTGCTACCCAAACCACTGAGAACGACAAGTCATCTCAGCAATAGGGGGGTGG
D V K H V N I N T A T Q T T E N D K S S Q Q *

→ Pol

TCTACCTGTCCCCAAATACCCTGAAACCATGGTATGTGTAGGGGATACAGTGGTACTAGCATTGATTGACACTGGAGCT
M V C V G D T V V L A L I D T G A
GAACATTCTGTAATAGATAAATGCTTAGTCCCAAAAGGGTTAAGACCAGCACACCACCAAAAACTACAAGGCCTTAATG
E H S V I D K C L V P K G L R P A H H Q K L Q G L N G
GTTCTCCAGTGTCATTTCCAGTCTTTAATTTAACTATTAATATCTCCAATACTGACATCCTTCTCAAATGTGCTGTGAA
S P V S F P V F N L T I N I S N T D I L L K C A V K
AGCAAACTTACAACCGTATAAACTTATAATCTCTCACCAAGAGGTAAAAGATTGCATCTTGCCTTTCTTCCCTCAGCCT
A N L Q P Y K L I I S H Q E V K D C I L P F F P Q P
CAAACTCAGGTCAAAGTTGTAAATATCATCACAACTGATGAGAGGCAAATTCATGTAAATACTCAATTAGCTGAAACAA
Q T Q V K V V N I I T T D E R Q I H V N T Q L A E T M
TGCTCTCACAAGCCAGCAAGCTTAAATTAAAAGCTTTACTGGAAAAACATTATGAGGTCCTCCAGAGGCATAAAAATCA
L S Q A S K L K L K A L L E K H Y E V L Q R H K N Q
AACAGGGTTTAGAGATATAGAACCTCACCAACTTAAAGGTAAAATCCACCCAGCTCAGAAGCAGTACCCAATTAATACC
T G F R D I E P H Q L K G K I H P A Q K Q Y P I N T
AAGGCCATTCCTTCTATTCAAGTGGTAATTAATGAATTACTCGAACAAGGAGTGCTGGTAAAGCAAACTAGTCCAACAA
K A I P S I Q V V I N E L L E Q G V L V K Q T S P T N
ATATGGCTGTGCTACCAGTGCCAAAACCTGATGGAACTTGGCGGTTGGTGCTAGACTATAGAGCCCTCAATAAACATTC
M A V L P V P K P D G T W R L V L D Y R A L N K H S
TGAACCAGTTAGGGCCCAGAACCAGCACTCCTCTGGTATTCTAGCCAACATTGAGAGAAAAGCTTATAAATCCTCTGTG
E P V R A Q N Q H S S G I L A N I E R K A Y K S S V
GATTTGGCAAATGGGTTCTGGTCTCATCCCATTAGGGAAGAAGACTGGCCCAAGATGGCTTTCACTTGGTGCGGCTTTC
D L A N G F W S H P I R E E D W P K M A F T W C G F Q
AGTATCTCTGGACACGCCTCCCCCAGGGATTTTTAAACAGTCCAGCATTGTTTTCTGCTGATGTAATATCACTGGTGGG
Y L W T R L P Q G F L N S P A L F S A D V I S L V G
ACAACTCCCAGGAGTATATTGCTATGTTGATGACATATACTTGACACATGACACTGAAGAGGAACACCTTAAAATTTTG
Q L P G V Y C Y V D D I Y L T H D T E E E H L K I L
GATCAAGTCTTAGAAATCCTCATCAAAGCTGGATATGTCATTAACATAAAGAAGTCCAAACTCTGTCGTAAAGTAGTGG
D Q V L E I L I K A G Y V I N I K K S K L C R K V V D
ATTTCCTGGGATTTTCCCTTTCTGATGAGGGGAGAGGCCTCTCAGATCAGTATAGGGAAAAACTTGCAGCTATTAAACC
F L G F S L S D E G R G L S D Q Y R E K L A A I K P
TCCTCAGACACTCAGACAGCTTCAATCTGTAATGGGGTTGTTGAATTTTTCAAGACTTTTTGTCAAAGGATTTTCTGAA
P Q T L R Q L Q S V M G L L N F S R L F V K G F S E
CTGGCTAAGCCTCTGTATGACCTCATCACAGTCAAGCCTTATCATTTCACCCCACAGGCTCATGAAGCACTAACTAAAC
L A K P L Y D L I T V K P Y H F T P Q A H E A L T K L
TATTAAGTGCAATTCAAACAGCCCCTAATCTCACCAATAGAGATAACACTAGAGCACTTGTAATTAGGATAGTTACAAG
L S A I Q T A P N L T N R D N T R A L V I R I V T S
CCCTAGATCAGCTGTAGCAGCTTATTACAATGTAGGGGATAAACTGCCCATTCAGTATGTCTCCTACAATTTTAGTCAA
P R S A V A A Y Y N V G D K L P I Q Y V S Y N F S Q
CCTGAAATGAAATTTTCCAAAATAGAGAAAGTCTGTGTTATGGTAAATATGGCATTAATAAAAGGAAGGGTATTAGCCC
P E M K F S K I E K V C V M V N M A L I K G R V L A H
ACGAACAAGATATTGAAATACACACTGCCATACCAGCTCTGCAGACTTACACCAGAGCCAGTGAACCTAATGCCAAGGC
E Q D I E I H T A I P A L Q T Y T R A S E P N A K A
ATTGGCAACCAGGTGGGATCTCTGGTGTACCCTATTCTCTGATCAAAGGTTGACTTTTAAGACAGTCACTAACATGCCT
L A T R W D L W C T L F S D Q R L T F K T V T N M P
GATCTCGATTGCCTACCACCCTTTGAGGATGAAGTGGCAGTAGCCAGTCCTCCTGCACCCATCCTTCCATTGGAACAAT
D L D C L P P F E D E V A V A S P P A P I L P L E Q Y
ATGTGGAAGTATATTATTCTGATGGCTCAGCCAAACGAAACCAACAAAAGAATTATGCAGGTATAGGGATAGTCAAAGG
V E V Y Y S D G S A K R N Q Q K N Y A G I G I V K G
AAAGTTTACTACTCCCACACATTTCGAGCCAGAAGAAACAAAAGCTGTACCATTGGGTCCAGCTTCAGCACAGTATGCT
K F T T P T H F E P E E T K A V P L G P A S A Q Y A
GAAGTCATGGCCTTACTGGATGCTGTTAAACAAGCAACAGATACTGGGCCAGTGCTTATATGCACAGACTCAGTATATG
E V M A L L D A V K Q A T D T G P V L I C T D S V Y A
CCCAAAGGGGATATACTGAAGATTTACACTATTGGGCTATAAGGAATTTCCATGACTCTAGGAATGCCAAACTCAAATA
Q R G Y T E D L H Y W A I R N F H D S R N A K L K Y
TGCCGACAAATGGAAACAACTAGATCAGCTTAAAAGGGACAAGCCTCTAGTAAGGGTAATACATGTGCCAGGGCATACG
A D K W K Q L D Q L K R D K P L V R V I H V P G H T

```
CCCGGAACTGTACATTCATGTGGAAATGGTTTAGCTGACTCATTGGCACAAGGTGCTGTAAAATGCCCTACAGTCAACG
P G T V H S C G N G L A D S L A Q G A V K C P T V N V
TGATTCTAACTAGAGCACAGGCCAGGACTCACATGGATAGTATTTTACAACAGTGTCTCGACCCAACCTGTCCAAACCC
 I L T R A Q A R T H M D S I L Q Q C L D P T C P N P
TCCTGGGTATCCTACTGCTTATCAGTATGATACCAATGAACATGGGCAATGTGTGGTCACAATGCCTGGAGGAACTTAT
 P G Y P T A Y Q Y D T N E H G Q C V V T M P G G T Y
GTCATACCTCCCATTCATGCCAGACCTGGGCTGATCAAGGAGGCCCATTGTGGCATTGGGCATATACATCTTGGGGGTA
V I P P I H A R P G L I K E A H C G I G H I H L G G N
ACAATACAGGCAAAACCTTGCGCCGCCTTTACTGGTGGCCAGGCCTATACACTGAGTGTAAAAGATACGTGGCTAACTG
 N T G K T L R R L Y W W P G L Y T E C K R Y V A N C
TCCTGAATGTTTGGCTGTTAATGTTACTCCCACTACTAGACCACCTAATTTACGCCCACCCAGAGGAGCCCTTTTTGAC
 P E C L A V N V T P T T R P P N L R P P R G A L F D
AAGATCTTTTTAGATTTTGTGGGCCCCCTTCCCCGCTCCAATGGCTATACTGCCATTTTGATTCTTGTCGAGTCCTTAT
K I F L D F V G P L P R S N G Y T A I L I L V E S L S
CCTCTTTTACCTGGTTACTCCCCTGCCGGGATCAGTCTGCGTCCACCACTGTGACTGCTCTTTCTGCCCGTTTCTTTCC
 S F T W L L P C R D Q S A S T T V T A L S A R F F P
GGCGTCTATTGAACCTAAATGTTTCCATTCTGACCAAGGGGGGGGGGACTTTACCTCCCAACTTTTCAAAAAAATGTGC
 A S I E P K C F H S D Q G G G D F T S Q L F K K M C
TCAGAACGGAACATACGGGTTGAATATAGCACTCCACACCATCCCCAGAGTGCTGGGGTGGTGGAAAGAAAGAACAGAG
S E R N I R V E Y S T P H H P Q S A G V V E R K N R G
GTCTCAAAGCAGCGCTCACTAAGCTGGTTCGCAACAGACCCCGCAAGTGGTTCCAGGTTCTAGACATAGTTCAAACAGG
 L K A A L T K L V R N R P R K W F Q V L D I V Q T G
GCTTAACAATACTCCAATAGCGAGGAATGAGCATGGGGCCACCCCATTCTTTCTGATGTATGGTCAACATATGAATACA
 L N N T P I A R N E H G A T P F F L M Y G Q H M N T
CCATTGACTAATGCTGAACCTAGTTTTGAACATCCAGATAGAATGGAGGCCCTTGCAGCACTACAAGAAATACGTGATC
P L T N A E P S F E H P D R M E A L A A L Q E I R D L
TAATGGCACAAGAGAGTAGAGGGATACAACAACAACTGCCAGAGTCCAATAGACTTTGGATCCCAAAGGTGGGAGAATG
 M A Q E S R G I Q Q Q L P E S N R L W I P K V G E W
GGTCCAAGAAAAAGCAGTTAGGGCCAATCCTAGCTTAAGGGATAAGTATCATAAGCCCACACAGATAATTGAAGTTTTG
 V Q E K A V R A N P S L R D K Y H K P T Q I I E V L
ACACCTAAGGCTGTGGTAATTGCTGCAACAGGAGCTAACCAACAACAACTTAGAAAAACTGTTTCTGTAGACAATCTAA
T P K A V V I A A T G A N Q Q Q L R K T V S V D N L K
                    → Env
AGAGAACGCCAGCTGACTACAATGGCAACAAACAAGTATCACCTAGCTTCCCAGAAGACTCAAGGGCCCCGTCCAGTGA
 R T P A D Y N G N K Q V S P S F P E D S R A P S S E
                    M A T N K Y H L A S Q K T Q G P R P V R
GACTGGAGGAAGTAAGGATACTACTAACCAACAGCAACAAGGACTCACCAGTGACCAGGTCTGCAGGCCCAAAGAAAAA
 T G G S K D T T N Q Q Q Q G L T S D Q V C R P K E K
 L E E V R I L L T N S N K D S P V T R S A G P K K K
                     Pol←
ACGCAAAAGAAAAGGAAAACCAAGTAAGGTGGGGGGTTGCTATAAATTCCTTATTGCTTTGTTGATAATGGCTGGACTC
T Q K K R K T K *
 R K R K G K P S K V G G C Y K F L I A L L I M A G L
ATAGCCCTAGGGACAGGTATCCTTAGGATACTTTGGGCCTATGCTGAAACCAAGATTCAGGTCCCAATGGAGGATAACA
I A L G T G I L R I L W A Y A E T K I Q V P M E D N N
ATTCAACCTACAATTATACTACTGGTCTGCACATGAGTCGAAGAACCTTGTCAAAAGGAGAAGACAATGAAACCCTGGA
 S T Y N Y T T G L H M S R R T L S K G E D N E T L E
ATGGCATTCCTTACCACTGGGGATGACAACCAGAGTAGTACCAATTCCATTGTCTTTGACCAGAGGCCTGGTTCAAATA
 W H S L P L G M T T R V V P I P L S L T R G L V Q I
CCAGTTAACTTAGTGCTGAATTCAAAACATTTGGCACAGGTTGCTGGGATTCAATCGGCCAAAGGAAGAAAGATGCTCA
P V N L V L N S K H L A Q V A G I Q S A K G R K M L S
GTAACAATTTGGTGCTACTTATGGAACAAGCCAAACTTCAAAATCCTTCCTTTGAGGTGGCTTCAGGATCACCAGAACA
 N N L V L L M E Q A K L Q N P S F E V A S G S P E Q
ACCTTCTTCATATTTAGCAGACCAGTGCTTTGCCGAATTAGGACATTGCTATCTGATTGAATATAATCAACCAAGAACT
 P S S Y L A D Q C F A E L G H C Y L I E Y N Q P R T
TTGCCTGATCCTTATATAATGGCAGATGAATGCTTAAGACCTGATGGCATTTGGCTAAGCAACAAAGGAATTCCACAAT
L P D P Y I M A D E C L R P D G I W L S N K G I P Q W
GGTATCTGGCAGCTGATCAATACACTGCACATACCCTGGAAGAAATCTTCAGAAAGGATCCTGATGCCAGGATGAGGGA
 Y L A A D Q Y T A H T L E E I F R K D P D A R M R E
ATACCGCATTCCTGGAGGGCGACAAACCTACACTGGAGCAATTATGTGTAGTCCATTACTCTACGGAAGCAAATGGAAA
 Y R I P G G R Q T Y T G A I M C S P L L Y G S K W K
GAAGAACAGCATCAATATGATGGAAGAGAGGAATTGGATATTCTTCTAAATGATTGCATGCAAAACCACAGCACTGGGT
E E Q H Q Y D G R E E L D I L L N D C M Q N H S T G L
```

```
TGTTAAAATCTAGTTGCCTCTCTCCCGAGTGGCATGTTAATGGTACCAAGGAAGGGAATATATTCCCTTACCTTATGGA
 L  K  S  S  C  L  S  P  E  W  H  V  N  G  T  K  E  G  N  I  F  P  Y  L  M  E
GCAAAACCATGATACCTTCTGTTCTATGCCCAGATTTCCCGCATTTTTAAACACCACCTTGTATCACGTAACTTGTGGT
Q  N  H  D  T  F  C  S  M  P  R  F  P  A  F  L  N  T  T  L  Y  H  V  T  C  G
GCTGCAGAAGTCAGAAACCTCACTAATGAGGAACAAAGTAAATGTGTTAATGATACTGTTGCCAAGGAGAAAGGTATAT
A  A  E  V  R  N  L  T  N  E  E  Q  S  K  C  V  N  D  T  V  A  K  E  K  G  I  Y
ACTGGGATTGTGTACGAGGTTGTGGGGGTAGAAAAATGGTGTATTGTGACATCAAAAATACAACCTGCTATTCAGAGAA
 W  D  C  V  R  G  C  G  G  R  K  M  V  Y  C  D  I  K  N  T  T  C  Y  S  E  K
ATATCCTACTACTCTTGGTAATAAAAGATTTTATGGGATACACAAAGGACCTCTCAGTATAATAAATAAGACAGTTATC
Y  P  T  T  L  G  N  K  R  F  Y  G  I  H  K  G  P  L  S  I  I  N  K  T  V  I
GACAGGCCAAATTATTCTGTATATTCTCTGTATCAGATCTGTAAGGACAGAGTTAAAATCTATCCCCTTACTGATGTAT
D  R  P  N  Y  S  V  Y  S  L  Y  Q  I  C  K  D  R  V  K  I  Y  P  L  T  D  V  L
TATCAGGATTGAAAAAATTGACTGTGGTCTCTAAAGGGAAATATGACTTCCCTTGCTCTAAGCGTACTAATTGTACTGA
 S  G  L  K  K  L  T  V  V  S  K  G  K  Y  D  F  P  C  S  K  R  T  N  C  T  D
TGTATATGTCCTGGATATGGTCCCTACTAAAGCCATTTGGGGAACTTATGTCACAATATCTGACTTAAACATAACTCAT
V  Y  V  L  D  M  V  P  T  K  A  I  W  G  T  Y  V  T  I  S  D  L  N  I  T  H
TCTAAACATGCAGGAAACTTTAATTTGACAAACATTCATGATAATTGTCCTCAGACGGCTGCCCAAGTGGGTGGCAACC
S  K  H  A  G  N  F  N  L  T  N  I  H  D  N  C  P  Q  T  A  A  Q  V  G  G  N  Q
AGAGGCGTCTGTTAGCGACAAATGATCAATACCTTACCCAATCAGCCACCCTTACGAATAAGGCTATTAACCTTCTTAG
 R  R  L  L  A  T  N  D  Q  Y  L  T  Q  S  A  T  L  T  N  K  A  I  N  L  L  S
CCAAACTATGGATGACAACTTTGCCCAATTGGAGCAAGGTTTTAAAGTGCTTCAAAGTAGCCTGATGCACACATTAAGG
Q  T  M  D  D  N  F  A  Q  L  E  Q  G  F  K  V  L  Q  S  S  L  M  H  T  L  R
GTACTTACTAAAGATATGGCAACTTTAGGAGATGCCATTAAAGTCAAACAAGTAGAAGCTCAGATACAGAGGGGATTGG
V  L  T  K  D  M  A  T  L  G  D  A  I  K  V  K  Q  V  E  A  Q  I  Q  R  G  L  A
CACTTTTAGAAAATAATAAGGTCCCTTGGGCACTCCTAAATATTTCTAATATCTTAGAAAGGTTCAATCAAAGTGCAGA
 L  L  E  N  N  K  V  P  W  A  L  L  N  I  S  N  I  L  E  R  F  N  Q  S  A  E
AATGAACGCTGTCATGCGTCATACTGCCACTGCCTATTTATATAATATCAAACAGGTTAAACAAGATTTCCACTACAAC
M  N  A  V  M  R  H  T  A  T  A  Y  L  Y  N  I  K  Q  V  K  Q  D  F  H  Y  N
CAGGCCTTGGATAATCCTACCTGGATGGTGAACCAAGAGAAGTATATTATGCAATTCATTGTACAAATGCAATTACCTG
Q  A  L  D  N  P  T  W  M  V  N  Q  E  K  Y  I  M  Q  F  I  V  Q  M  Q  L  P  V
TAACAGGAGGAATTTACTATAAAGTGTGGGAGATTTACAACCCTGGCTTTCTAGTAAAAACTAATAACTTATTGGCATT
 T  G  G  I  Y  Y  K  V  W  E  I  Y  N  P  G  F  L  V  K  T  N  N  L  L  A  L
AGCTAAAGTAAGGCCTCCTTACCAGTATATTGCTAAAGATAAATGTGGCAACATAAATTACCTTAAAGTAAAGGAATGC
A  K  V  R  P  P  Y  Q  Y  I  A  K  D  K  C  G  N  I  N  Y  L  K  V  K  E  C
TATTTAAGAGGATTTAGATATTGTGATAATTGGGAAAAGCAATTGCCCTGTGGAAAAGAACCACAGACAACTCAATCAT
Y  L  R  G  F  R  Y  C  D  N  W  E  K  Q  L  P  C  G  K  E  P  Q  T  T  Q  S  C
GTCCGATCACGGTAAAACCAATCTATTCTAGAACTTTTGCACAGGTTACCTCTTTAGGAAATGGCTCTTATATAATTAT
 P  I  T  V  K  P  I  Y  S  R  T  F  A  Q  V  T  S  L  G  N  G  S  Y  I  I  M
GGTATCCTACTCTGAATGCGACATTCCCAGTATGAGAACTGTTATAGTCACCCCAAGCACCCGAATGACTTGTTACAAT
V  S  Y  S  E  C  D  I  P  S  M  R  T  V  I  V  T  P  S  T  R  M  T  C  Y  N
ATGACCTTGTTCCCCCTTAATGTGTCACGAGTAAATGCTATGGCAGAGGAGGGAGTCACACCATTCCATCTTGACTTCC
M  T  L  F  P  L  N  V  S  R  V  N  A  M  A  E  E  G  V  T  P  F  H  L  D  F  P
CAATTCTCGTTGCAGTCATTGCACGCTTGCAAGATTATGAAATTACACTCTCATATACTCATGACAACATAACTCAGCT
 I  L  V  A  V  I  A  R  L  Q  D  Y  E  I  T  L  S  Y  T  H  D  N  I  T  Q  L
CTTGGCACGCTCTAGGCAATTGCTTTCAATGGTGAGATTTGAAGCAGCTGAAATACCACAATGGCTACAGCTGATAGGG
L  A  R  S  R  Q  L  L  S  M  V  R  F  E  A  A  E  I  P  Q  W  L  Q  L  I  G
GAAGGACTAATTGAGCTCATACCACAAGTCAAGAGTGCTATAGAATGGGGCATAGACTTGGCAGCTAAGACTATAACAC
E  G  L  I  E  L  I  P  Q  V  K  S  A  I  E  W  G  I  D  L  A  A  K  T  I  T  Q
AGACAGTAACAAGTGTTTTGCAATCATTAAACTTTCTAGTACCTGTTTTGATAATAGCAGGTGTAGTCTTTGTGCTAAT
 T  V  T  S  V  L  Q  S  L  N  F  L  V  P  V  L  I  I  A  G  V  V  F  V  L  I
                                        Env ←

     → ORF1
TTTAGTACTCAAGTTTTTTTCTCAGAAAGATGGATCCTCATAAGCTGATAAAAGAAAGTCTCAGCATTAAACCAGAGAT
L  V  L  K  F  F  S  Q  K  D  G  S  S  *
    Y  S  S  F  F  L  R  K  M  D  P  H  K  L  I  K  E  S  L  S  I  K  P  E  M
                                                                       ORF2→
GGTATTGAGGAGATTTCTGAAGATAGAACAGATGATCCTGTCGAAACCTGTTGAAAATCACTCACGTGCACGTATAAAG
V  L  R  R  F  L  K  I  E  Q  M  I  L  S  K  P  V  E  N  H  S  R  A  R  I  K
                                                                            S
                                                                    ORF2 ←
TGGTGTGAAAGGAAACAAGACTTTGTTGTGTATAACGACTGGAATTTGGGATTGTTATCGCATAGAGGACATTGTTGAA
W  C  E  R  K  Q  D  F  V  V  Y  N  D  W  N  L  G  L  L  S  H  R  G  H  C  *
G  V  K  G  N  K  T  L  L  C  I  T  T  G  I  W  D  C  Y  R  I  E  D  I  V  E
```

```
AGTATAAAGCCGCTTATATTCATAGGGCAGAGCCTAAATCTAAAGACTGATAAACACCTGCCAATTCCAGCTGTTTATG
S  I  K  P  L  I  F  I  G  Q  S  L  N  L  K  T  D  K  H  L  P  I  P  A  V  Y  V
TGCCCGAGGGCTACTGCTCCCGTTTGGCATACATATATCATATTTTTGATATGGATAAAGGAGCTTCAGCTCAACAAAA
  P  E  G  Y  C  S  R  L  A  Y  I  Y  H  I  F  D  M  D  K  G  A  S  A  Q  Q  N
TTACACTCCAATGAAAAGAACCTGGAAGGACAACTATGAGAATGATTCTCCACCTTCTTATAAATCCTTATTGAGGATG
 Y  T  P  M  K  R  T  W  K  D  N  Y  E  N  D  S  P  P  S  Y  K  S  L  L  R  M
CATGCTCTGCGCAGTTTACCCACTCAGAATTACTTCTCTTCGCATATCAATACTAAGAGATGCTGTTGTTATTTAAAAC
H  A  L  R  S  L  P  T  Q  N  Y  F  S  S  H  I  N  T  K  R  C  C  C  Y  L  K  H
                             ORF2←

                                                                           LTR
ATACATGTTATGGCACCATTAACCACACTCTTTAAATAAGAATATTTTTCCTGCCCGGGGGGGGGGGGTTAATGTACTAG
  T  C  Y  G  T  I  N  H  T  L  *
TAGAGGCTCCTTTTCCTACTGTCAGTTTAGCTTGTCATATAATTGATGTCCTAAACCTATTGGCATGAAAAGATCAGCA
ATCCAAGGTTAATTGCTTGCATGGAACTGACGAATGTGTTATTTACAAGTAGAAGCTTCTAGACCTCCTGTGTACAGCC
TAATTGTCACAGCTGATACTGTTATGCTAACATTTAAGGTGACAGGAAAAATATTTGTTAAGTTTTGTCCTAGACTAAA
GAAATGTAAAATTATATTATCTTCCTGCACATCCTGTGTAGGACCAAGCCTTAGAGCCAGAATATTGTATAAATACAAC
ACCTAGGTCCATAGCATGTCAGAACGATCAGAATAATCTTCTCAGCAGAGAGACTGAGGGTGAGAGCTGCTGGTTGCAG
ACTCTATTGGACTAGGTGAGCACTGTTACTATCCTTCTATATTCTCAGATTGTGAGTTCTGGCCTAGTCCCTTTATGCT
TAAGTAAGATTGATATATTGAAATTGCTAGCCTTTGTAATTTTATACTATAAACCACTCTAGAGCTAATTGTATGATTT
ATATATATCATTAATAAATATATAACATTTTAAGGACCACTCAAGCCTCTCTTTTAATTCATTATTACTAGAAATATAT
GAATCTTTATTTACTGGTGATTTAGCAATTATCAAGAGGGAGGTAAGCGGACCTACTCCTTTGATGTACTAAAGGACGG
TTGCCTAGTACTAATTCAATGCCATCCCTATAATAGAGTAAAAGTATTCAATTAGGCAGGCAATACTGCTCCACATCAC
AG
```
