# Supplementary material for: An Endogenous Foamy-like Viral Element in the Coelacanth Genome
Source: PLoS Pathog. 2012 Jun 28;8(6):e1002790. doi: 10.1371/journal.ppat.1002790 (PMC3386198; doi:10.1371/journal.ppat.1002790)
Supplement: Figure S3 — Phylogenetic relationships among retroviruses. The phylogeny was reconstructed with the Bayesian method via MrBayes 3.1.2. The posterior probabilities are shown on the nodes. The foamy virus and lentivirus clades were highlighted in red and blue, respectively. Branch lengths are in expected amino acid changes per site. (PDF) [file ppat.1002790.s009.pdf]

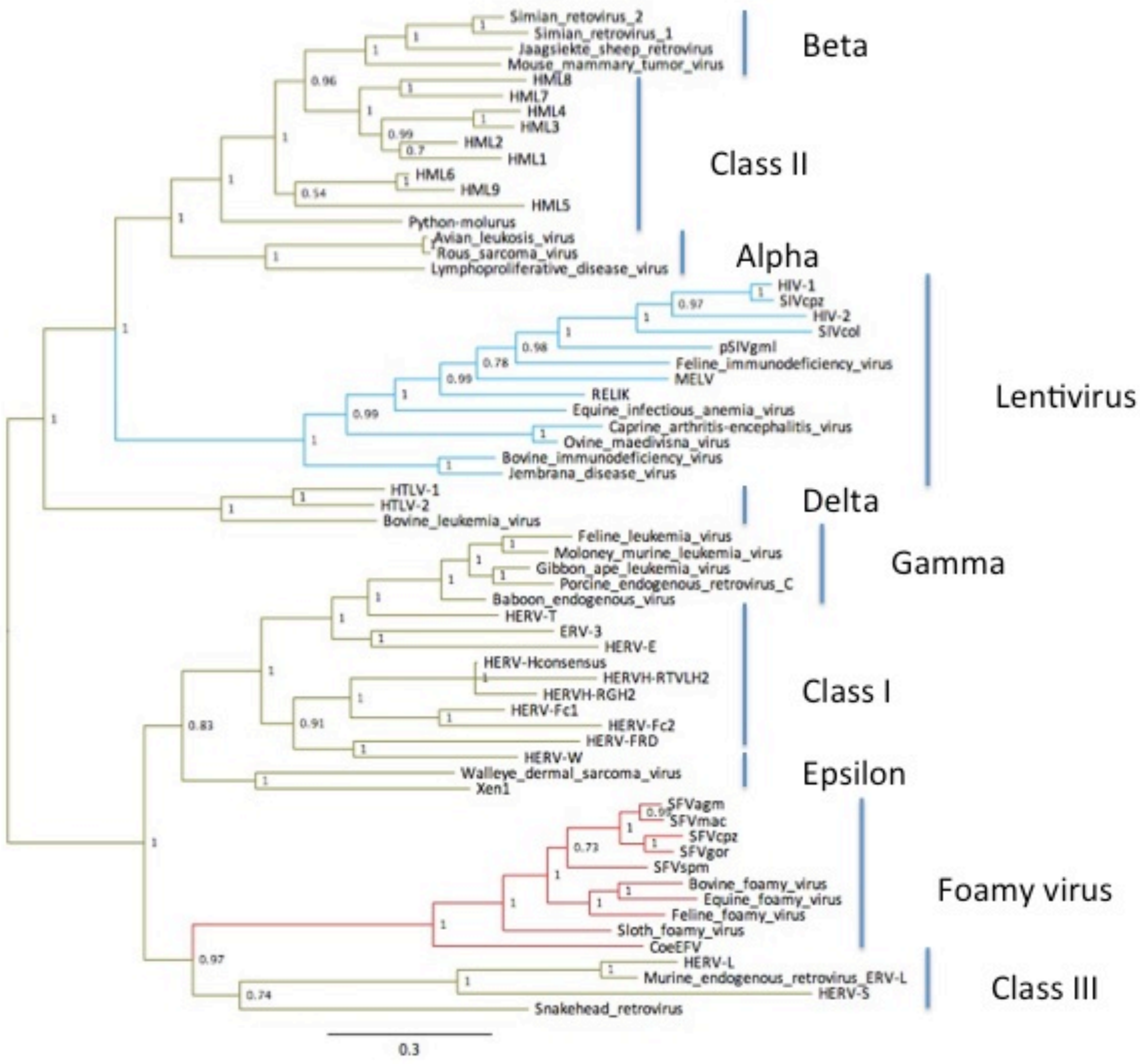

Figure S3. Phylogenetic relationships among retroviruses. The phylogeny was reconstructed with the Bayesian method via MrBayes 3.1.2. The posterior probabilities are shown on the nodes. The foamy virus and lentivirus clades were highlighted in red and blue, respectively. Branch lengths are in expected amino acid changes per site.
