# Supplementary material for: An Endogenous Foamy-like Viral Element in the Coelacanth Genome
Source: PLoS Pathog. 2012 Jun 28;8(6):e1002790. doi: 10.1371/journal.ppat.1002790 (PMC3386198; doi:10.1371/journal.ppat.1002790)
Supplement: Figure S4 — Alignment of the two sets of sequences used for dating CoEFV invasion. Flanking sequences are shown for each sequence set with consensus genomic sequence of CoeEFV. (PDF) [file ppat.1002790.s010.pdf]

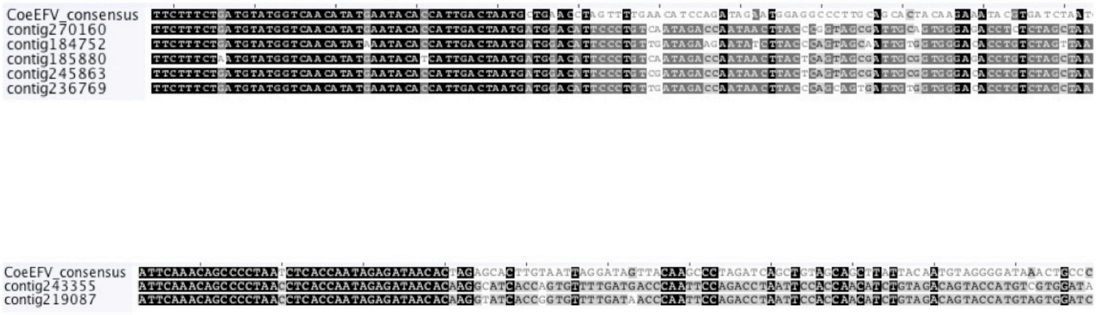

Figure S4. Alignment of the two sets of sequences used for dating CoEFV invasion. Flanking sequences are shown for each sequence set with consensus genomic sequence of CoeEFV.
