# Supplementary material for: An Endogenous Foamy-like Viral Element in the Coelacanth Genome
Source: PLoS Pathog. 2012 Jun 28;8(6):e1002790. doi: 10.1371/journal.ppat.1002790 (PMC3386198; doi:10.1371/journal.ppat.1002790)
Supplement: Figure S6 — A) Midpoint rooted phylogenetic tree of foamy viruses. The phylogeny is the 50% majority-rule consensus tree inferred from conserved region of foamy virus Env protein alignment with MrBayes 3.1.2. Posterior probabilities are shown at the nodes. Branch lengths are in expected amino acid changes per site. B) A plot of the correlation between foamy virus divergence and their vertebrate hosts' divergence times. The virus branch lengths are derived from the virus tree in A. (PDF) [file ppat.1002790.s012.pdf]

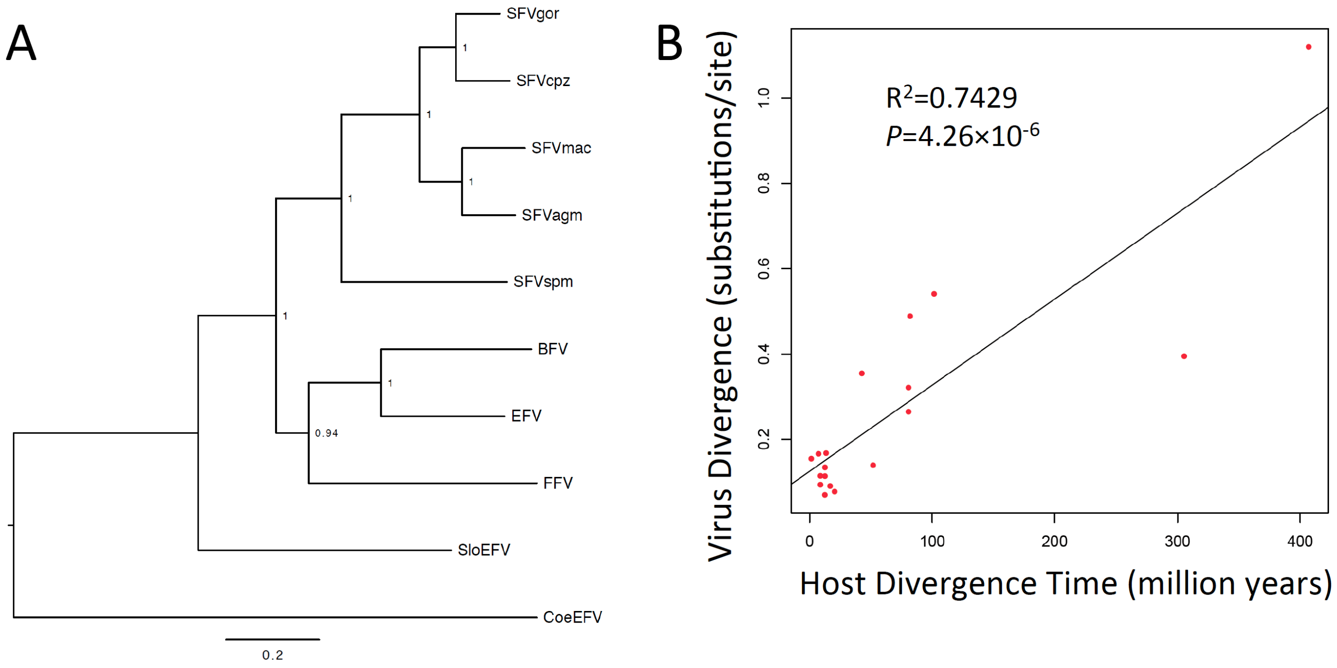

Figure S6. A) Midpoint rooted phylogenetic tree of foamy viruses. The phylogeny is the 50% majority-rule consensus tree inferred from conserved region of foamy virus Env protein alignment with MrBayes 3.1.2. Posterior probabilities are shown at the nodes. Branch lengths are in expected amino acid changes per site. B) A plot of the correlation between foamy virus divergence and their vertebrate hosts' divergence times. The virus branch lengths are derived from the virus tree in A.
