# Supplementary material for: An Endogenous Foamy-like Viral Element in the Coelacanth Genome
Source: PLoS Pathog. 2012 Jun 28;8(6):e1002790. doi: 10.1371/journal.ppat.1002790 (PMC3386198; doi:10.1371/journal.ppat.1002790)
Supplement: Table S1 — The representative retrovirus sequences used for genome screening and phylogenetic reconstruction. (PDF) [file ppat.1002790.s013.pdf]

Table S1. The representative retrovirus sequences used for genome screening and phylogenetic reconstruction

Name	Accession No./Source
Bovine foamy virus	NC 001831.1
Equine foamy virus	NC_002201.1
Feline foamy virus	NC_001871.1
SFVspm	EU010385.1
SFVgor	HM245790.1
SFVcpz	NC_001364.1
SFVmac	NC_010819.1
SFVagm	NC_010820.1
Bovine leukemia virus	NP_056895.1
Human T-cell leukemia virus type 1 (HTLV-1)	NP_057860.1
Human T-cell leukemia virus type 2 (HTLV-2)	NP_041003.2
Bovine immunodeficiency virus	NP_040563.1
Human immunodeficiency virus type 2 (HIV-2)	NP_663784.1
Human immunodeficiency virus type 1 (HIV-1)	ABK51636.1
Simian immunodeficiency virus of colobus (SIVcol)	AAK01033.1
Simian immunodeficiency virus of chimpanzee (SIVcpz)	ABU53017.1
Jembrana disease virus	AAA64389.1
Caprine arthritis-encephalitis virus	NP_040939.1
Ovine maedivisna virus	YP_536867.1
Equine infectious anemia virus	NP_056902.1
Feline immunodeficiency virus	NP_040973.1
Lymphoproliferative disease virus	AAA62195.1
Avian leukosis virus	YP_004222728.1
Rous sarcoma virus	NP_056886.1
Murine endogenous retrovirus ERV-L	CAA73251.1
Python molurus endogenous retrovirus	AAN77283.1
Mouse mammary tumor virus	NP_056880.1
Jaagsiekte sheep retrovirus	NP_041186.1
Simian retrovirus 2	AAA47562.1
Simian retrovirus 1	AAA47732.1
Snakehead retrovirus	NP_043924.1
Walleye dermal sarcoma virus	NP_045937.1
Feline leukemia virus	NP_955577.1
Moloney murine leukemia virus	NP_057933.2
Baboon endogenous virus	BAA89659.1
Gibbon ape leukemia virus	NP_056790.1
Porcine endogenous retrovirus C	CAC39617.1
Rabbit endogenous lentivirus type K (RELIK)	Ref. 1
Gray mouse lemur prosimian immunodeficiency virus (pSIVgml)	Ref. 2
Sloth endogenous foamy virus (SloEFV)	Ref. 3
Coelacanth endogenous foamy virus (CoEFV)	This study
Mustelidae endogenous lentivirus (MELV)	Ref. 4
HERV-S	Ref. 5

HERV-L	Ref. 5
HERV-FRD	Ref. 5
HERVH-RTVLH2	Ref. 5
HERV-Hconsensus	Ref. 5
HERVH-RGH2	Ref. 5
HERV-Fc1	Ref. 5
HERV-Fc2	Ref. 5
HERV-W	Ref. 5
HERV-E	Ref. 5
ERV-3	Ref. 5
HERV-T	Ref. 5
Xen1	Ref. 5
HML1	Ref. 5
HML2	Ref. 5
HML3	Ref. 5
HML4	Ref. 5
HML5	Ref. 5
HML6	Ref. 5
HML7	Ref. 5
HML8	Ref. 5
HML9	Ref. 5

References

1. Katzourakis A, Tristem M, Pybus OG, Gifford RJ. (2007). Discovery and analysis of the first endogenous lentivirus. Proc Natl Acad Sci USA 104: 6261-6265.
2. Gifford RJ, Katzourakis A, Tristem M, Pybus OG, Winters M, Shafer RW. (2008). A transitional endogenous lentivirus from the genome of a basal primate and implications for lentivirus evolution. Proc Natl Acad Sci USA 105: 20362-20367.
3. Katzourakis A, Gifford RJ, Tristem M, Gilbert MT, Pybus OG. (2009). Macroevolution of complex retroviruses. Science 325: 1512.
4. Han GZ, Worobey M. (2012). Endogenous lentiviral elements in the weasel family (Mustelidae). Mol Biol Evol (in press).
5. Jern P, Sperber GO, Blomberg J. (2005). Use of endogenous retroviral sequences (ERVs) and structural markers for retroviral phylogenetic inference and taxonomy. Retrovirology 2: 50.
