# Supplementary material for: An Endogenous Foamy-like Viral Element in the Coelacanth Genome
Source: PLoS Pathog. 2012 Jun 28;8(6):e1002790. doi: 10.1371/journal.ppat.1002790 (PMC3386198; doi:10.1371/journal.ppat.1002790)
Supplement: Table S2 — The matching contigs identified in coelacanth genome. (PDF) [file ppat.1002790.s014.pdf]

Table S2. The matching contigs identified in coelacanth genome

Contig Number	Genomic Regions Represent
contig243355	5'LTR-*gag*-*pol*
contig219087	5'LTR-*gag*-*pol*
contig210236	5'LTR-*gag*-*pol*
contig187426	*gag*-*pol*
contig036414	5'LTR-*gag*-*pol*
contig236767	5'LTR-*gag*-*pol*
contig184753	5'LTR-*gag*-*pol*
contig185881	*gag*-*pol*
contig184751	*env*-ORF1/2
contig187425	*pol*-*env*-ORF1/2-3'LTR
contig274525	5LTR-*gag*
contig178313	*pol*-*env*
contig247568	*pol*
contig270160	*pol*
contig184752	*pol*
contig236768	*pol*
contig185880	*pol*
contig279762	*pol*-*env*-ORF1/2
contig245863	*pol*
contig236769	*pol*
contig281509	*pol*-*env*-ORF1/2
contig241846	*env*-ORF1/2
contig111507	*env*-ORF1/2
contig236770	*env*-ORF1/2
